# Transient power equalization control strategy of virtual synchronous generator in isolated island microgrid with heterogeneous power supply

**DOI:** 10.1038/s41598-023-39121-6

**Published:** 2023-08-03

**Authors:** Changwei Gao, Yongchang Sun, Weiqiang Zheng, Wei Wang

**Affiliations:** 1 College of Electrical and Automation Engineering, Liaoning Institute of Science and Technology, Benxi, 117004 China; 2 Dalian Economic and Technological Development Zone Heat Supply Co., LTD, Dalian, 116600 China; 3 Yingkou Power Supply Company, Yingkou, 115002 China

**Keywords:** Power stations, Electrical and electronic engineering

## Abstract

In the parallel supply system of synchronous generator and virtual synchronous generator, the physical structure and control structure of the two kinds of power supply are quite different, and it is difficult to distribute the transient power of the two kinds of power supply evenly when the load changes abruptly. Especially in the case of sudden load increase, virtual synchronous generator bears too much load in the transient process because of its fast adjustment speed, and even causes short-term overload, which makes the capacity of virtual synchronous generator can not be fully utilized. In view of this problem, firstly, the mechanism of transient power uneven distribution of two heterogeneous power sources is explained from the differences of frequency modulation, voltage regulation and output impedance. Secondly, virtual speed regulation, virtual excitation and dynamic virtual impedance are added to the traditional virtual synchronous generator control to simulate the speed regulation characteristics and electromagnetic transient characteristics of the synchronous generator, so as to realize the transient and steady-state power equalization between heterogeneous power supplies when the virtual synchronous generator and the synchronous generator run in parallel. Thirdly, in order to ensure the fast dynamic response characteristics of the virtual synchronous generator in independent operation mode, the traditional virtual synchronous generator control algorithm is still maintained in independent operation mode, and the mode switching control link based on state tracking is designed to realize smooth switching between the two working modes. Finally, the hardware in loop experiment results based on RT-LAB show that the proposed control method can realize the transient and steady-state power equalization when the virtual synchronous generator and the synchronous generator operate in parallel, it can keep the fast voltage regulation and frequency modulation ability when the virtual synchronous generator operates independently, and can realize smooth switching between independent and parallel operation modes.

## Introduction

Parallel power supply of synchronous generator (SG) and inverter is widely used in various independent power systems^1,2^, such as island and remote mountain power supply system, ship power system and so on. Constant power control, constant voltage/frequency control, droop control, virtual synchronous generator (VSG) control are commonly used in inverters. Among them, constant power control does not participate in the voltage adjustment and frequency adjustment of the system, and is only suitable for the grid-connected operation of inverters^3^. Constant voltage/frequency inverter is usually used as the main control power supply of constant voltage and constant frequency in microgrid^4^, providing voltage reference and frequency support for independent microgrid. By simulating the primary frequency modulation and primary voltage regulation characteristics of SG, sag control can realize the active and reactive power equalization of multiple parallel inverters without interconnection lines^5–8^. VSG control is based on droop control, and the simulation of SG mechanical motion and electromagnetic relationship is added, which can solve the problem of insufficient inertia and damping of distributed generation. Since it was put forward, it has been widely concerned by scholars at home and abroad^9–11^.

In the parallel power supply system of synchronous generator and inverter, the inverter adopts VSG control strategy and is connected to the system in the form of voltage source, which can provide inertia for the system and participate in the power adjustment of the system according to the set droop characteristics. It can avoid the complicated switching process of traditional constant voltage/frequency control and constant power control and the problems of reverse power of generator caused by sudden reduction of load, this allows the inverter to plug and play and autonomously coordinate operation^12^. Aiming at the problem of steady-state power equalization between inverters based on droop control and VSG control, the research has been mature at present, and the control methods based on virtual impedance, virtual capacitance and line impedance identification have achieved good control results^13–15^. After appropriate improvement, these methods can also be applied to parallel systems between inverters and synchronous generators, but VSG and SG cannot achieve transient power equalization during sudden load increase and reduction, and VSG may have overcurrent faults due to taking most of the loads in transient process. In order to ensure the safe operation of the system, the inverter will reserve a certain amount of transient reserve capacity, which makes the inverter capacity can not be fully utilized.

In^16^, for isolated island microgrid with SG and VSG, transient virtual impedance is introduced to prevent VSG overcurrent when load suddenly increases, but the problem of uneven distribution of transient power is not solved. On the basis of analyzing the output impedance of synchronous generator, a transient power sharing control method based on improved dynamic virtual impedance is proposed in^17^, which has certain effect on improving transient power sharing. However, the influence of synchronous generator speed regulation and excitation dynamics on power uneven sharing is not considered, and transient power distribution is still different. In^18^, a dynamic sag coefficient control method based on proportional and high-pass filter term is proposed, which improves transient power averaging in parallel operation. However, the introduction of dynamic sag coefficient deteriorates the power supply quality and frequency and voltage regulation ability of inverter. In^19^, the mechanism of transient active power distribution between diesel generator and virtual synchronous generator in isolated island microgrid is analyzed. By introducing phase shift control and active power response delay into VSG control, the transient active power sharing effect is improved, but the transient reactive power sharing and the influence of voltage change caused by reactive power on transient active power sharing are not considered in the analysis process. In^20^, the causes of uneven transient power distribution between droop control inverter and synchronous generator is analyzed. By introducing Parker equation of synchronous generator and speed regulation and excitation links, a control strategy of inverter which precisely simulates synchronous generator is proposed. In^21^, a control method for simulating diesel generator is put forward, which makes the inverter consistent with the conventional diesel generator model, speed regulating system and voltage regulating system in terms of control order, logic, parameters and so on. However, the mechanism of uneven transient power distribution has not been studied in detail in^20^ and^21^, and the improved control strategy is based on the complex mathematical model of synchronous generator, which requires accurate physical parameters of synchronous generator. The control strategy is complex and the control flexibility is not high.

On the basis of revealing the mechanism of transient power unevenness between VSG and SG, this paper introduces virtual speed regulation, virtual excitation, dynamic virtual impedance into the traditional VSG control algorithm, which effectively solves the problem of transient power unevenness when VSG and SG run in parallel due to the difference of speed regulation characteristics and electromagnetic transient characteristics. In order to take into account the ability of fast frequency modulation and voltage regulation under independent operation mode, a mode switching control is designed to keep the traditional VSG control in independent operation mode, and virtual speed regulation, virtual excitation and dynamic virtual impedance are added in parallel operation mode, so that the independent and parallel operation modes can be smoothly switched and the requirements of independent operation and parallel operation can be met at the same time. Hardware in loop test based on RT-LAB controller verifies the effectiveness of the proposed control strategy.

## Description of transient power inequality between VSG and SG

### Introduction of VSG control principle

**Figure 1:**
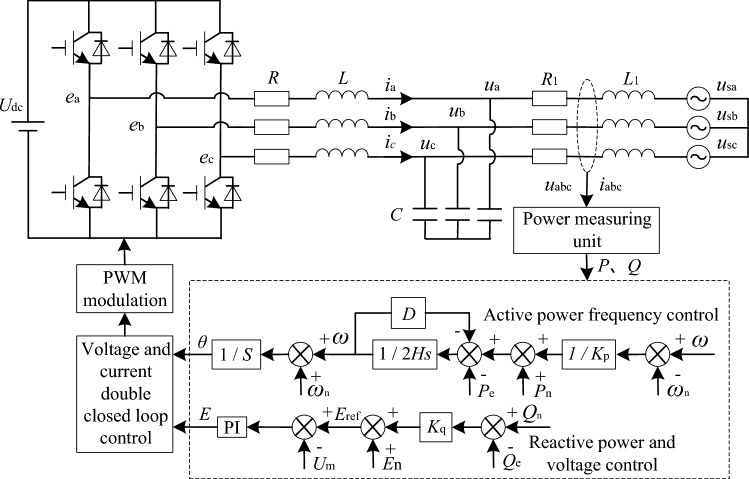
Traditional VSG topology and control system structure.

The VSG topology and control block diagram are shown in Fig. 1, where *C*, *L* and *R* are filter capacitor, bridge arm filter inductor and internal resistance respectively, *L*_1_ and *R*_1_ are the load side filter inductance and internal resistance, respectively.

In Fig. 1, ignoring the filter capacitor, the frequency domain transfer function of the VSG main circuit can be expressed as:

1 $$ u_{{\text{o}}} = \frac{1}{{LCs^{2} + RCs + 1}}e{ - }\frac{Ls + R}{{LCs^{2} + RCs + 1}}i = G_{u} (s)e - G_{Z} (s)i $$

**Figure 2:**
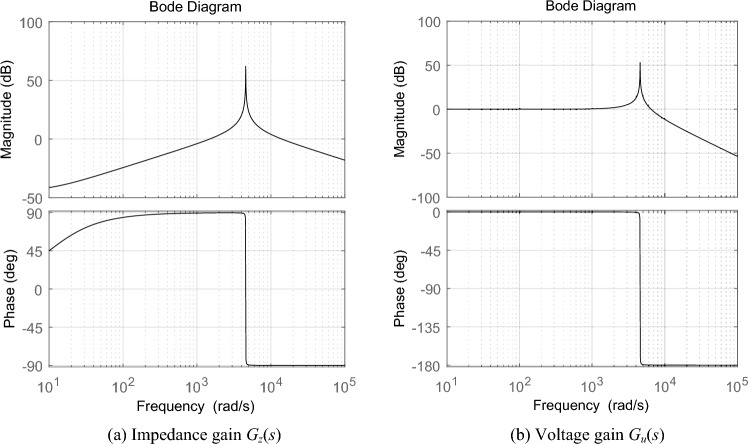
Bird diagram of impedance gain *G*_*z*_(*s*) and voltage gain *G*_*u*_(*s*) of VSG.

According to the design principle of filter circuit, the filter circuit parameters in VSG main circuit are selected as *L* = 0.6mH, *C* = 80μF and *R* = 6 mΩ. The Bird diagram of impedance gain *G*_*z*_(*s*) and voltage gain *G*_*u*_(*s*) is shown in Fig. 2. As shown in Fig. 2a, the output impedance of VSG shows inductive characteristics in the fundamental and low-order harmonic frequency bands. Near the resonant frequency, the output impedance amplitude is amplified. It shows capacitive characteristics in high frequency band. As shown in Fig. 2b, the voltage gain of VSG is 1 in fundamental and low harmonic frequency bands. Near the resonant frequency, the amplitude of harmonic voltage is seriously amplified. In high frequency band, the amplitude of harmonic voltage decays rapidly.

Based on above analysis, the relationship between the fundamental component of VSG output voltage *u*_o_ and its modulated signal *e* is described as follows:

2 $$ u_{{\text{o}}} = \left[ \begin{gathered} u_{a} \hfill \\ u_{b} \hfill \\ u_{c} \hfill \\ \end{gathered} \right] = \left[ \begin{gathered} e_{a} \hfill \\ e_{b} \hfill \\ e_{c} \hfill \\ \end{gathered} \right] - jX\left[ \begin{gathered} i_{a} \hfill \\ i_{b} \hfill \\ i_{c} \hfill \\ \end{gathered} \right] = E_{ref} \left[ \begin{gathered} \cos (\omega t) \hfill \\ \cos (\omega t - 120^{0} ) \hfill \\ \cos (\omega t + 120^{0} ) \hfill \\ \end{gathered} \right] - jX\left[ \begin{gathered} i_{a} \hfill \\ i_{b} \hfill \\ i_{c} \hfill \\ \end{gathered} \right] $$

where *E*_ref_ is the amplitude of the modulated signal, and *X* is the equivalent reactance value of VSG output impedance *G*_*z*_(*s*) in fundamental frequency band.

In summary, VSG equates the bridge arm voltage of grid-connected inverter to the excitation induced electromotive force of traditional synchronous generator stator winding. The impedance of filter circuit is equivalent to synchronous reactance of synchronous generator, and the filtered output voltage is equivalent to stator winding terminal voltage.

VSG control uses inverter to simulate the running characteristics of synchronous generator to provide the moment of inertia and damping needed for stable operation of the system, and has the functions of primary frequency modulation and voltage regulation, which is mainly composed of active power–frequency and reactive power-voltage control links. Active power–frequency link is used to simulate the primary frequency modulation and rotor motion equation of synchronous generator. In the standard value system, the rated capacity and rated voltage of inverter are taken as reference values, active power frequency droop characteristics and rotor motion equation are shown in (3).

3 $$ \left\{ {\begin{array}{*{20}l} {P_{{\text{m}}} = P_{{\text{n}}} + \frac{1}{{K_{{\text{P}}} }}(\omega - \omega_{{\text{n}}} )} \hfill \\ {2H\frac{{{\text{d}}\omega }}{{{\text{d}}t}} = P_{{\text{m}}} - P_{{\text{e}}} - D(\omega - \omega_{{\text{n}}} )} \hfill \\ {\frac{{{\text{d}}\theta }}{{{\text{d}}t}} = \omega \omega_{{\text{b}}} } \hfill \\ \end{array} } \right. $$

where *P*_n_ is the rated active power of VSG, *K*_p_ is the active-frequency sag coefficient, *P*_m_ and *P*_e_ are the virtual mechanical power and the output active power of the virtual synchronous generator. When the number of pole pairs is 1, the electrical angular velocity *ω* of synchronous generator is its mechanical angular velocity. *ω*_n_ is the rated angular velocity, *ω*_b_ is the reference value of angular velocity. *θ* is the virtual rotor angle, *H* is the time constant of virtual inertia, *D* is the virtual damping coefficient.

The reactive power-voltage control link mainly realizes reactive power-voltage sag control, and the output voltage amplitude command *E*_ref_ is obtained according to the current output reactive power and the given sag relationship.

4 $$ E_{{{\text{ref}}}} = E_{{\text{n}}} + K_{{\text{q}}} (Q_{{\text{n}}} - Q_{{\text{e}}} ) $$

where *E*_n_ is the rated voltage, *K*_q_ is reactive power sag coefficient, *Q*_n_ is the rated reactive power of VSG, *Q*_e_ is the reactive power of the VSG output. The voltage amplitude *U*_m_ feedback from the filter capacitor is introduced, and the internal potential *E* is obtained after PI link control, so that the VSG output voltage can quickly track the voltage command.

#### Synchronous generator control and characteristics

**Figure 3:**
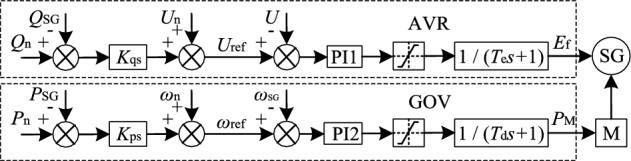
Speed control and exception control block diagram of synchronous generator.

Generator control includes prime mover governor (GOV) and automatic voltage regulator (AVR). For the convenience of analysis, the GOV and AVR of synchronous generators are simplified by single closed-loop PI control, and the prime mover and its speed regulation characteristics and exciter dynamic characteristics are equivalent by first-order inertia links respectively. The control block diagram is shown in Fig. 3, *K*_ps_ and *K*_qs_ are the active power–frequency and reactive power-voltage sag coefficients of synchronous generators respectively. *T*_d_ is the time constant of the first order inertia link of the prime mover and its speed regulating mechanism. *T*_e_ is the time constant of the first order inertia link of exciter. *E*_f_ is the generator excitation voltage*. P*_M_ is the input mechanical power of prime mover.

### Transient power unevenness between VSG and SG

**Figure 4:**
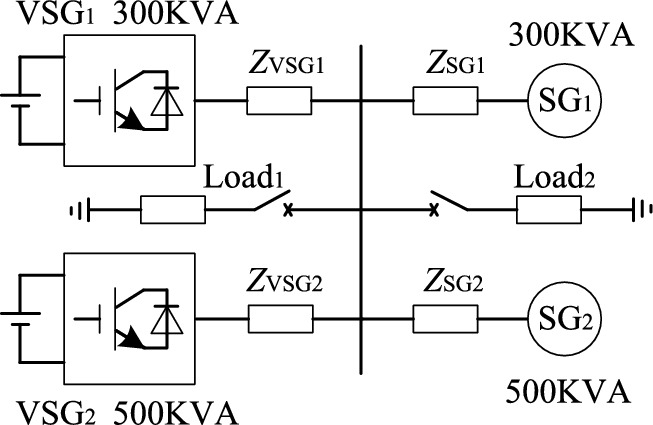
Simulation system topology.

The physical and control structures of the two kinds of power supply are different. When the load increase or decrease suddenly, VSG and SG can’t realize transient power sharing equally. Build the simulation model as shown in Fig. 4, and the system equipment parameters are shown in Table 1 and Table 2.

**Table 1:** Virtual synchronous generator electronic and control parameters.

Parameter	VSG_1_	VSG_2_
DC voltage/V	560	560
Rated voltage/V	380	380
Rated capacity/KVA	300	500
Rated frequency/Hz	50	50
Filter inductor *L*/μ H	150	200
Filter capacitor *C*/μ F	300	300
Filter inductor *L*_1_/μ H	120	80
Frequency-active equivalent sag coefficient 1/*K*_p_ + *D*	100	100
Active power–frequency sag coefficient *K*′_p_	0.01	0.01
Reactive power and voltage sag coefficient *K*_q_	0.03	0.03
Inertial time constant *H*/s	2.2	2.2
Virtual damping coefficient *D*′	0.15	0.25

**Table 2:** Synchronous generator electronic and control parameters.

Parameter	SG_1_	SG_2_
Rated capacity/KVA	300	500
Rated voltage/V	380	380
Power factor	0.8	0.8
Rated frequency/Hz	50	50
*X*_d_	1.8	2.0
*X'*_d_	0.21	0.18
*Xʺ*_d_	0.16	0.14
*X*_q_	0.95	1.05
*Xʺ*_q_	0.19	0.17
*T'*_d_	0.38	0.40
*Tʺ*_d_	0.016	0.019
*Tʺ*_q_	0.014	0.018
Inertial time constant *H*/s	2	2.5
Damping coefficient *D*	0.15	0.2
*K*_ps_	0.01	0.01
*K*_qs_	0.02	0.02
Speed regulating ring *K*_P2p_	75	75
Speed regulating ring *K*_P2i_	150	150
Excitation ring *K*_P1p_	4	4
Excitation ring *K*_P1i_	60	60

**Figure 5:**
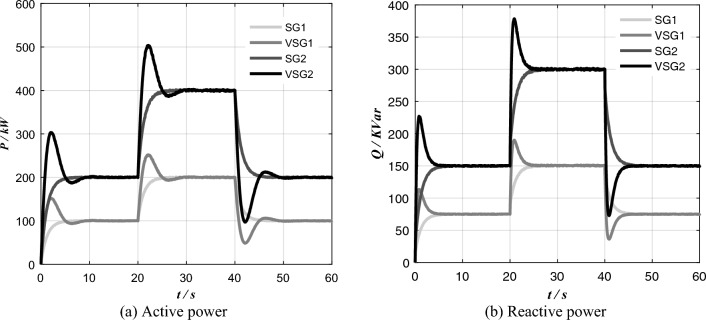
Simulation results of load mutation in parallel operation of traditional VSG and SG.

Simulation conditions: initially, two VSG and two SG are put into operation at the same time with 600 + j450 kVA resistance inductance load; when t = 20 s, the resistive and inductive load of 600 + j450 kVA suddenly increases; when t = 30 s, the resistive and inductive load of 600 + j450 kVA is suddenly reduced. As shown in Fig. 5, the simulation results show that there is a large overshoot in the active and reactive power output of each VSG. This kind of current overshoot in transient process may lead to inverter overcurrent. If a part of inverter capacity is reserved to ensure that overcurrent hazard does not occur during transient loading, the inverter capacity will not be fully utilized.

## Mechanism of uneven distribution of transient power

In order to suppress the output active power oscillation in grid-connected VSG without affecting the steady-state output power, an improved VSG control strategy based on transient electromagnetic power compensation is proposed in this section.

### Equivalent circuit model

The output voltage frequency and amplitude of synchronous generator are determined by prime mover governor and excitation controller, respectively. The equivalent output impedance of synchronous generator is the sum of q-axis reactance *X*_q_ and line impedance. During the transient process, q-axis is affected by the sub-transient process of damping winding, and the sub-transient reactance *X*′_q_ of q-axis transients rapidly to synchronous reactance *X*_q_.

In VSG control, voltage and current double closed-loop control has high bandwidth, and its output signal can track the given value quickly, so its influence is ignored in this paper. VSG is equivalent to a controlled voltage source with ideal AC power supply and impedance in series. Low-voltage transmission lines generally present resistivity, but inverter can make the output line present inductance by configuring isolation transformer and designing virtual impedance.

**Figure 6:**
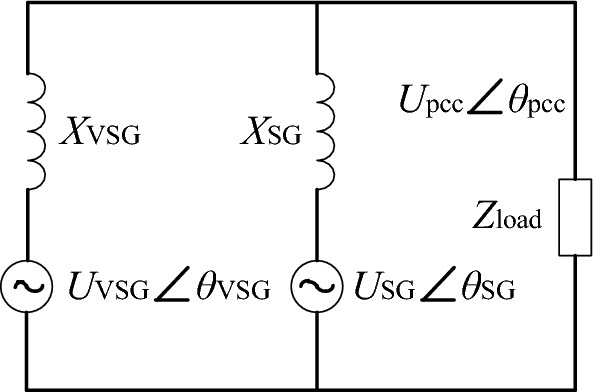
Equivalent circuit of VSG and SG parallel system.

Establish the equivalent circuit of VSG and SG parallel power supply shown in Fig. 6. *U*_VSG_ is the output phase voltage amplitude of reactive power-voltage control loop in VSG, *θ*_VSG_ is the phase generated by virtual rotor motion equation in VSG control algorithm, *X*_VSG_ is the sum of load side filter reactance, transmission line reactance and virtual reactance. *U*_SG_ is the transient electric potential *E* '_q_ of the synchronous generator, *θ*_SG_ is determined by the rotation angle of the synchronous generator, and *X*_SG_ is the sum of the generator output reactance and the transmission line reactance.

### Difference analysis of active power–frequency control link

According to the equivalent circuit model shown in Fig. 6, the line impedance is purely inductive, ignoring the influence of voltage amplitude change, the output voltage of VSG and SG and the voltage amplitude of common point are considered to be constant. The expressions of VSG and SG output active power are derived and analyzed by small signal:

5 $$ \Delta P_{{\text{k}}} = \frac{3}{2}\frac{{U_{{\text{k}}} U_{{{\text{pcc}}}} }}{{X_{{\text{k}}} }}\cos \delta_{{{\text{k0}}}} \Delta \delta_{{\text{k}}} = \frac{{K_{{{\text{ck}}}} \omega_{{\text{n}}} }}{s}(\Delta \omega_{{\text{k}}} - \Delta \omega_{{{\text{pcc}}}} ) $$

where the subscript “k” represents VSG or SG, *δ*_k_ is the phase difference between the output voltage of VSG or SG and the voltage of the common connection point, *δ*_k_ = ∫(*ω*_k_-*ω*_pcc_)*ω*_n_d*t*, *U*_pcc_ and *ω*_pcc_ are the phase voltage amplitude and angular frequency of the common connection point respectively, *K*_ck_ = 3*U*_k_*U*_pcc_cos*δ*_k0_/2*X*_k_.

According to the control block diagram and (5), the transfer function relationship between VSG output power and angular frequency of common connection point is represented as (6), the transfer function relationship between SG output power and angular frequency of common connection point is described as (7). *K*_P2p_ and *K*_P2i_ in (7) are the proportional and integral parameters of PI2 module in Fig. 2 respectively; *H*_SG_ and *D*_SG_ are inertia time constant and damping coefficient of generator respectively.

6 $$ \frac{{\Delta P_{{{\text{VSG}}}} }}{{\Delta \omega_{{{\text{pcc}}}} }} = - \frac{{(2Hs + 1/K_{{\text{p}}} + D)K_{{{\text{cVSG}}}} \omega_{{\text{n}}} }}{{2Hs^{2} + (1/K_{{\text{p}}} + D)s + K_{{{\text{cVSG}}}} \omega_{{\text{n}}} }} $$

7 $$ \frac{{\Delta P_{{{\text{SG}}}} }}{{\Delta \omega_{{{\text{pcc}}}} }} = - \frac{{K_{{{\text{cSG}}}} (2H_{{{\text{SG}}}} \omega_{{\text{n}}} T_{{\text{d}}} s^{3} + 2H_{{{\text{SG}}}} \omega_{{\text{n}}} s^{2} - K_{{{\text{P2p}}}} \omega_{{\text{n}}} s - K_{{{\text{P2i}}}} \omega_{{\text{n}}} )}}{{2H_{{{\text{SG}}}} T_{{\text{d}}} s^{4} + (2H_{{{\text{SG}}}} + D_{{{\text{SG}}}} T_{d} )s^{3} + As^{2} + Bs + C}} $$

where $$A = D_{{{\text{SG}}}} + K_{{{\text{P2p}}}} + K_{{{\text{cSG}}}} \omega_{{\text{n}}} T_{{\text{d}}}$$, $$B = K_{{{\text{P2i}}}} + K_{{{\text{cSG}}}} \omega_{{\text{n}}} (K_{{{\text{pSG}}}} K_{{{\text{P2p}}}} + 1)$$, $$C = K_{{{\text{cSG}}}} \omega_{{\text{n}}} K_{{{\text{pSG}}}} K_{{{\text{P2i}}}}$$.

Set Δ*P*_VSG_/Δ*ω*_pcc_ = *G*_VSG_ and Δ*P*_SG_/Δ*ω*_pcc_ = *G*_SG_, based on Δ*P*_VSG_ + Δ*P*_SG_ = Δ*P*_load_, the following relationship can be obtained.

8 $$ \Delta P_{{{\text{VSG}}}} (1 + \frac{{G_{{{\text{SG}}}} }}{{G_{{{\text{VSG}}}} }}) = \Delta P_{{{\text{load}}}} $$

9 $$ \Delta P_{{{\text{SG}}}} (1 + \frac{{G_{{{\text{VSG}}}} }}{{G_{{{\text{SG}}}} }}) = \Delta P_{{{\text{load}}}} $$

The transfer functions between VSG and SG output power and load power in parallel operation can be obtained from (8) and (9), respectively.

**Figure 7:**
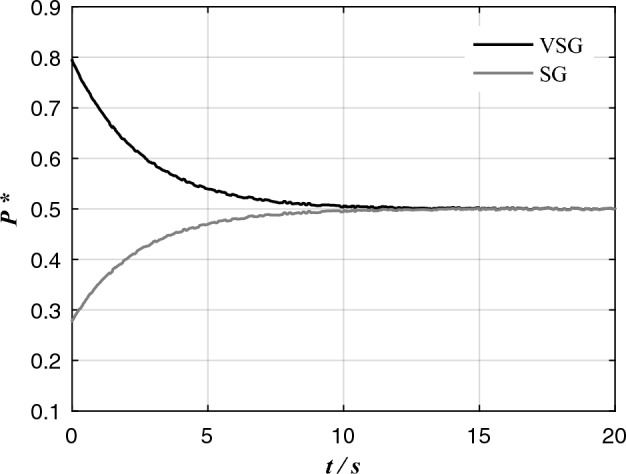
Step response of VSG and SG output power to active load.

Considering the relevant parameters of VSG_1_ and SG_1_ in Fig. 4, the influence of the difference of active power–frequency control links on transient active power distribution is mainly analyzed here. Considering the influence of output impedance on power distribution in the initial stage of transient process, *X*_SG_ = *X*ʺ_q_ + *X*_l_SG1_. The step response of VSG and SG output power to load is shown in Fig. 7, VSG output power can respond to load changes quickly, resulting in VSG bearing most of the load during transient process.

### Difference analysis of reactive power-voltage control link

According to the third-order mathematical model of synchronous generator, the following relation can be obtained.

10 $$ E_{{\text{q}}}{\prime} = (E_{{{\text{fd}}}} - (X_{{\text{d}}} - X_{{\text{d}}}{\prime} )I_{{\text{d}}} )\frac{1}{{T_{{{\text{d0}}}}{\prime} s + 1}} $$

where *T*′_d0_ is the d-axis transient open-circuit time constant, *E*_fd_ is the stator excitation electric potential, *E*′_q_ is the q-axis transient electric potential, *X*_d_ is the d-axis synchronous reactance of synchronous generator, *X*′_d_ is the d-axis transient reactance of synchronous generator.

Equation (10) shows that there is a first-order inertia link between SG transient electric potential *E*′_q_ and excitation voltage, which makes the output voltage unable to respond to the change of reactive load immediately, and armature reaction will also have an impact on excitation voltage regulation. In VSG control algorithm, the output variable *E* of reactive power-voltage control link is the output port voltage of inverter, which is equivalent to the amplitude of internal electric potential of VSG, and does not involve the simulation of electromagnetic transient process of excitation winding. In the parallel power supply system of VSG and SG, VSG can quickly respond to the change of reactive load and bear most reactive load in transient process.

### Output impedance difference analysis

During the transient process, the equivalent output impedance of the synchronous generator transients quickly from the sub-transient reactance *X*ʺ_q_ to the q-axis synchronous reactance *X*_q_. In the per unit system of synchronous generator, the value range of *X*_q_ is 1.0 ~ 2.3, while the filter reactance of low-voltage inverter is generally 0.2 ~ 1, and the output impedance of VSG only needs to consider the filter reactance of load side. The output impedance of SG is much larger than that of VSG. The output impedance difference determines the power distribution in the initial power frequency periods of load mutation, and is approximately inversely proportional to the output impedance^22^, so VSG will bear more loads in the initial stage of load mutation.

## Improved algorithm for transient power equalization

### Improve control algorithm and process

To realize transient power equalization of VSG and SG parallel power supply system, virtual speed control, virtual excitation control and dynamic virtual impedance are introduced into the traditional VSG control algorithm during parallel operation. At the same time, in order to ensure the fast voltage regulation and frequency modulation ability of VSG when it runs independently, it switches to the traditional VSG algorithm when it runs independently. A mode switching control strategy based on state tracking is designed, which ensures that the input voltage reference values of frequency and voltage outer loop in the two modes are consistent at switching time, prevents the impact caused by sudden change of phase and voltage reference values during switching, and realizes smooth switching between independent and parallel operation modes.

**Figure 8:**
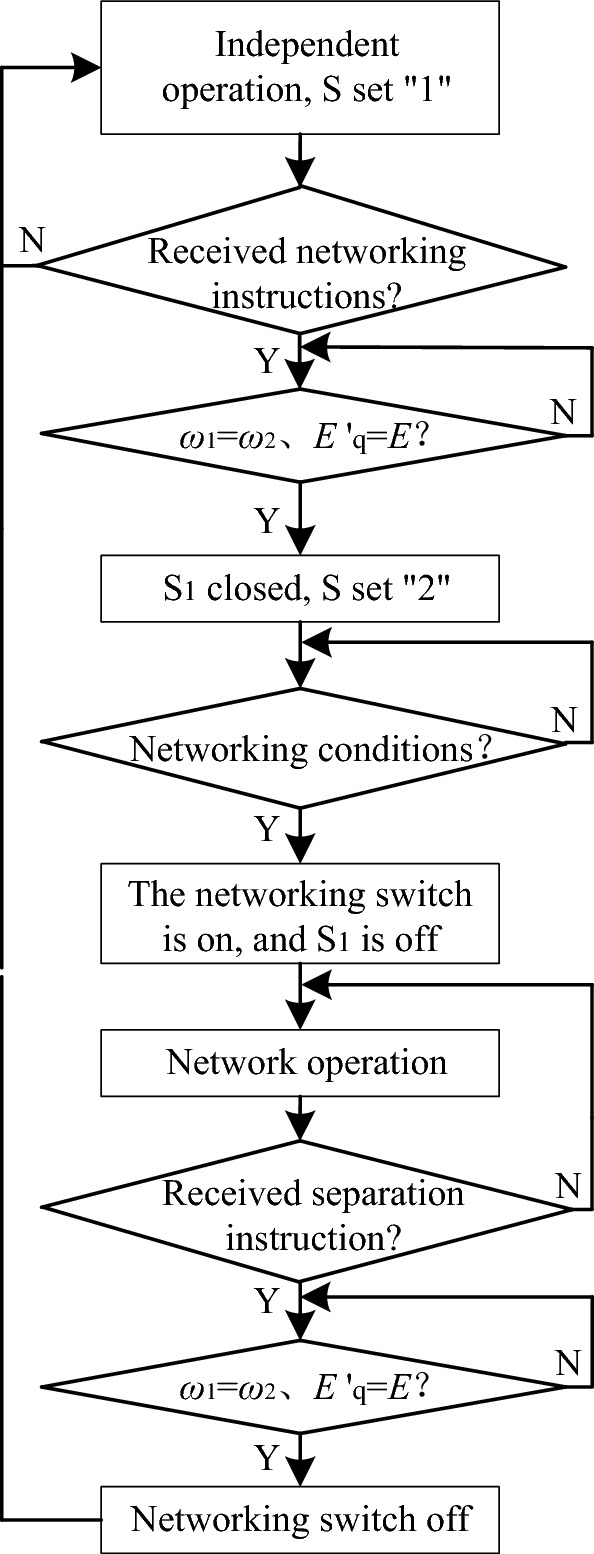
Operation process of improved control algorithm.

**Figure 9:**
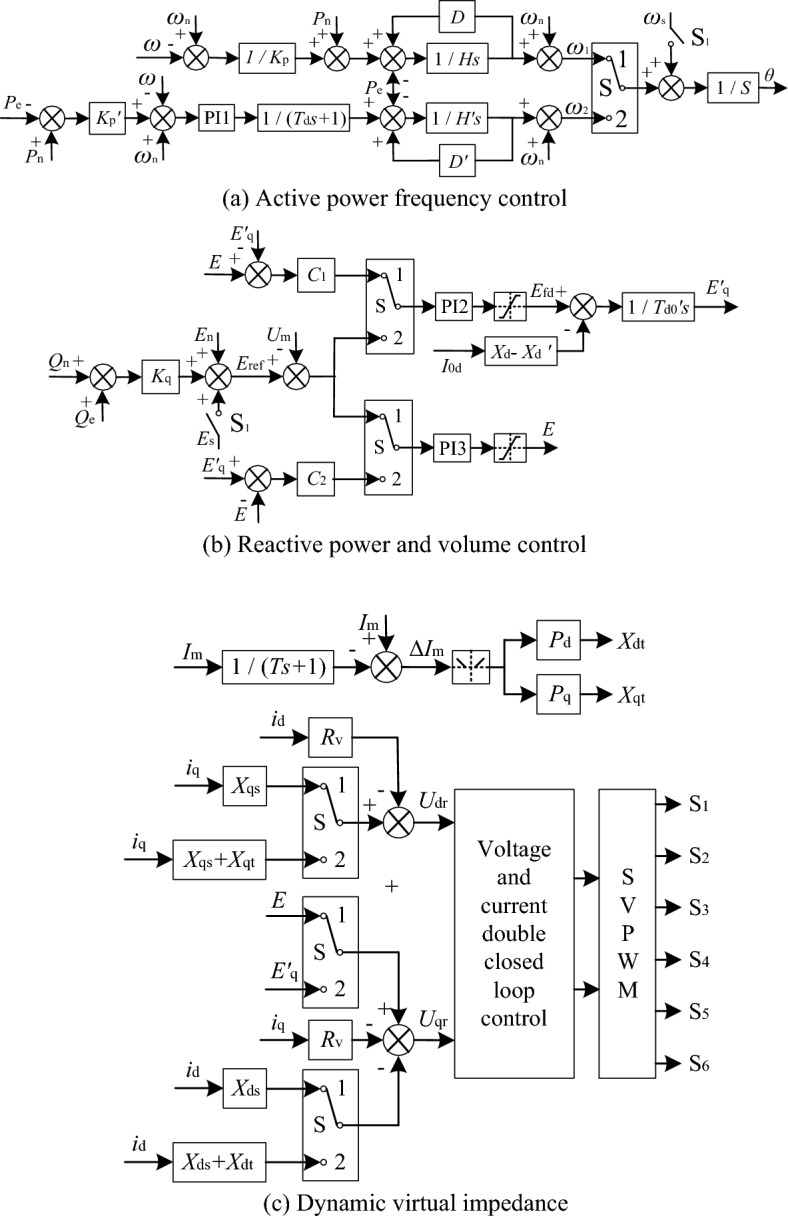
Improved VSG control method.

The operation flow chart is shown in Fig. 8, and the improved control algorithm is shown in Fig. 9. When running independently, the switch S is set to “1”. When receiving the networking instruction, if *ω*_2_ = *ω*_1_ and *E*′_q_ = *E*, all mode switching switches S are switched to “2”, and the pre-synchronization control switch S_1_ is closed at the same time, check whether the amplitude, phase and frequency of voltage on both sides of the grid meet the networking conditions, if the conditions are met, the closing operation is completed, and the pre-synchronization switch S_1_ is disconnected. When receiving the separation instruction, if *ω*_2_ = *ω*_1_ and *E*′_q_ = *E*, all the switching switches S are switched to “1”, and then the separation is performed. The following is a detailed introduction to each improved control link.

### Improvement of active power–frequency control link

The improved active power–frequency control link is shown in Fig. 9a. When running independently, S is set to “1”, and the active power–frequency control link keeps the traditional VSG control. When the network is running in parallel, S is set to “2”, the dynamic response of the speed control link of prime mover and the equivalent inertia link of the speed control mechanism can be simulated. In steady-state operation, the output frequency and power of the two modes meet the droop relationship, and the output frequency values of positions “1”and “2” are the same, which can ensure that the phase before and after switching does not change abruptly.

The design principle of related parameters is as follows: In traditional VSG control, the virtual damping coefficient *D* affects the droop characteristics of the control, and 1/*K*_p_ + *D* is the equivalent frequency-active droop coefficient. However, in the improved control method, the virtual damping coefficient *D*' no longer affects the droop control effect, and 1/*K*'_p_ is the equivalent frequency-active droop coefficient, which determines the steady-state power sharing between VSG and SG, and should be matched with the active-frequency droop coefficient *K*_ps_ in SG governor. *T*_d_ is set with reference to the equivalent inertia time constant of synchronous generator speed regulating mechanism. The virtual inertia time constant *H* ' and virtual damping coefficient *D* ' are consistent with SG, so that the output power response characteristics of VSG are consistent with SG.

### Improvement of reactive power–voltage control link

The improved reactive power-voltage link is shown in Fig. 9b, and the reactive power-voltage sagging link maintains the relationship of (4) when operating independently and in parallel. Switching control is introduced into the inverter output voltage tracking control link after droop link. When running independently, all switching switches S are set to “1”, keeping the traditional VSG control algorithm, and the output internal electrical potential amplitude instruction *E* is directly obtained by PI3 control link, and *E* is taken as the reference value of control variable *E*′_q_, and *E*′_q_ virtual tracking *E* is realized by PI2 control link. During network operation, S is set to “2”, VSG introduces the electromagnetic transient process of synchronous generator excitation winding through (9), *E*′_q_ is the output internal electrical potential amplitude of VSG in parallel operation mode, where *T*′_d0_, *X*_d_ and *X*′_d_ are set with reference to the standard value parameters of parallel SG, *E*′_q_ is taken as the reference value of control variable *E*, and *E* virtual tracking *E*′_q_ is realized through PI3 control link.

The *E*′_q_ and *E* variable tracking control links ensure that the internal electrical potential is consistent during switching and reverse switching, and smooth switching can be realized between modes. The parameters of PI2 and PI3 control links in Fig. 9 are adjusted according to VSG operation characteristics. In order to make the control parameters shared with *E* and *E*′_q_ tracking links, the proportional coefficients *C*_1_ and *C*_2_ are multiplied after *E*′_q_ and *E* feedback links respectively to adapt to PI control parameters.

### Dynamic virtual impedance design

Virtual impedance technology can change the output impedance characteristics of inverter and reduce the difference of output impedance characteristics between VSG and SG. In order to ensure that the voltage reference value does not change suddenly when the operation mode is switched, on the basis of the same phase and internal electrical potential amplitude, it is still necessary to ensure that the virtual impedance in the two modes is equal at the switching time. If the design of virtual impedance parameters matches the output impedance of SG, the larger virtual impedance will deteriorate the dynamic characteristics of VSG when it runs independently. Therefore, this paper proposes a dynamic virtual impedance algorithm, which can not only ensure that there is no impulse current at the time of mode switching, but also provide a larger virtual impedance during the load mutation process in the parallel operation mode of the network, and improve the problem of uneven distribution of transient power between VSG and SG caused by mismatch of output impedance. VSG virtual impedance is described as (11).

11 $$ \left\{ \begin{gathered} U_{{{\text{dr}}}} = 0 - R_{{\text{v}}} I_{{\text{d}}} + X_{{{\text{qv}}}} I_{{\text{q}}} \hfill \\ U_{{{\text{qr}}}} = E_{{\text{q}}}{\prime} - R_{{\text{v}}} I_{{\text{q}}} + X_{{{\text{dv}}}} I_{{\text{d}}} \hfill \\ \end{gathered} \right. $$

where *R*_v_ is the virtual resistance, *X*_dv_ and *X*_qv_ are d-axis and q-axis virtual reactance respectively.

The dynamic virtual impedance design method is shown in Fig. 9c, *R*_v_ is generally small, and the set values in the two modes are consistent. D-axis virtual reactance and q-axis virtual reactance consist of constant term and dynamic variation, respectively.

12 $$ \left\{ \begin{gathered} X_{{{\text{dv}}}} = X_{{{\text{ds}}}} + X_{{{\text{dt}}}} \hfill \\ X_{{{\text{qv}}}} = X_{{{\text{qs}}}} + X_{{{\text{qt}}}} \hfill \\ \end{gathered} \right. $$

Constant terms *X*_ds_ and *X*_qs_ are virtual reactance in steady state, which are used to improve power distribution at load abrupt change time. The transient variations *X*_dt_ and *X*_qt_ are introduced under the load abrupt change condition, and the output current amplitude variation Δ*I*_m_ of VSG is used to judge the load abrupt change condition. Δ*I*_m_ is obtained by the difference between the output instantaneous current amplitude *I*_m_ and the current amplitude after it passing through the first-order inertia link, the first-order inertia time constant *T* is set as the virtual inertia time constant *H*′. When Δ*I*_m_ exceeds the set threshold *ε*, it is judged as a load abrupt change condition, and the absolute value of the part exceeding the threshold value is multiplied by the proportional coefficients *P*_d_ and *P*_q_ respectively to obtain the dynamic changes *X*_dt_ and *X*_qt_ of the virtual impedance. Taking *X*_qt_ as an example, the expression is represented as (13).

13 $$ X_{{{\text{qvt}}}} = \left\{ \begin{array}{ll} 0, &\quad \left| \Delta I_{{\text{m}}}  \right| < \varepsilon   \\ P_{{\text{q}}} (1 - \varepsilon )\left| \Delta I_{{\text{m}}}  \right|,&\quad \left| \Delta I_{{\text{m}}}  \right| > \varepsilon \end{array} \right. $$

If the value of *ε* is too small, the dynamic virtual impedance will intervene when the disturbance is small, which is not conducive to the stability of the system. If the value is too large, the load mutation condition cannot be effectively detected. In the standard value system, *ε* is generally 0.08. The proportional coefficients *P*_d_ and *P*_q_ are flexibly set with reference to the ratio of the equivalent output impedance of SG to the equivalent output impedance of VSG.

When heterogeneous power supply operating independently, the switch S is set to “1” and the virtual impedance is a constant. When heterogeneous power supply running in parallel, the switch S is set to “2”, and the dynamic virtual impedance is introduced to improve the transient power distribution. The dynamic change of the virtual impedance under steady-state conditions is 0, and the virtual impedance is the same as that under independent operation, which can ensure smooth switching between modes.

## Experiment verification

**Figure 10:**
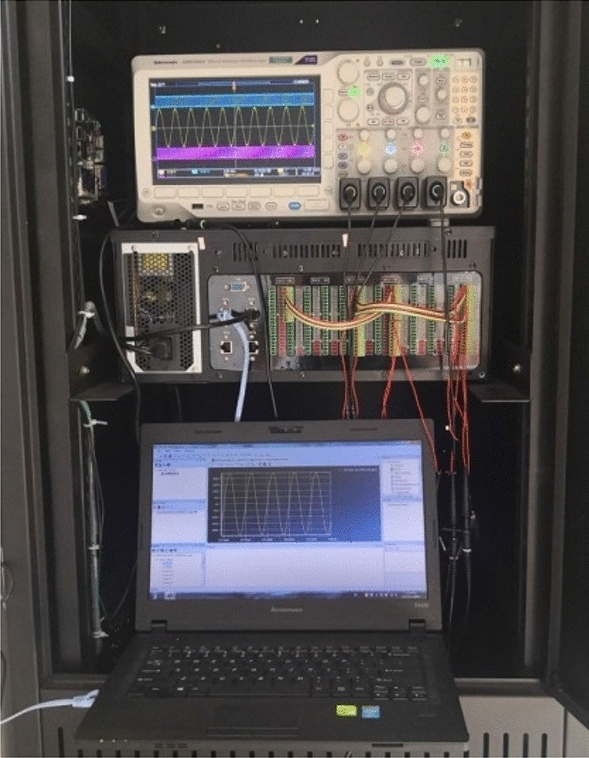
Hardware in loop experience system based on RT-LAB.

Based on RT-LAB real-time simulator (HBUREP-100) and DSP controller (TMS320F28335), a hardware-in-loop test platform is constructed to verify the effectiveness of the proposed control strategy. As shown in Fig. 10, the platform consists of upper computer, HBUREP-100 real-time simulator, controller, oscilloscope and other parts. The model of VSG and SG parallel system is built in the upper computer. The C code compiled from the model is loaded into the HBUREP-100 simulator to run, and the analog electrical signal and switching digital signal needed by the inverter control algorithm are output, and the control signal input by the controller is received. The controller collects the analog signal and digital signal output by HBUREP-100 simulator and realizes the control algorithm.

The VSG_1_ + SG_1_ parallel system in the power supply system shown in Fig. 4 is established. The experimental conditions are as follows: At the beginning, the synchronous generator is fully loaded and the inverter is empty; When t = 15 s, VSG networking instruction is issued, and the heterogeneous power supply network runs; When t = 30 s, the resistive and inductive load of 240 + j180 kVA suddenly increased; When t = 45 s, the resistive and inductive load of 240 + j180 kVA suddenly reduced; When t = 60 s, SG was cut off and the load was transferred to VSG.

**Figure 11:**
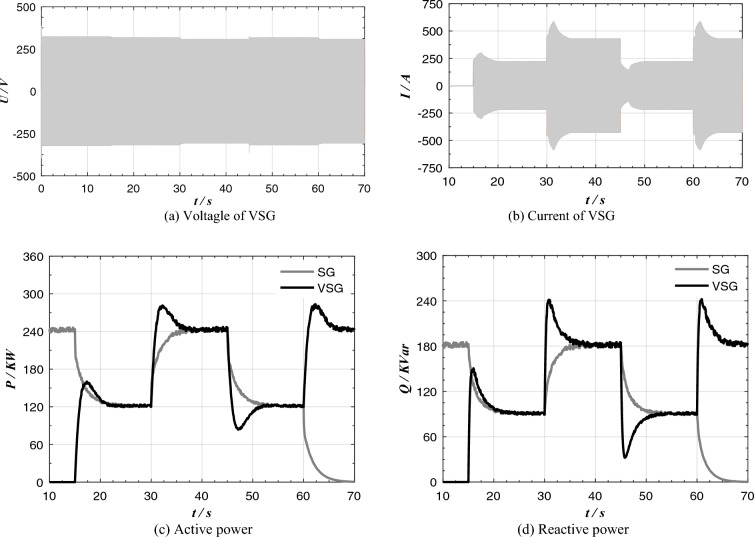
Experimental results of parallel operation of traditional VSG and SG.

The experimental results of parallel operation of traditional VSG and SG network are shown in Fig. 11, the output active power, reactive power and output current of VSG have large overshoot. When t = 30 s, the load surge makes the maximum active power overshoot per unit reach 0.15 and the maximum reactive power overshoot per unit reach 0.21.

**Figure 12:**
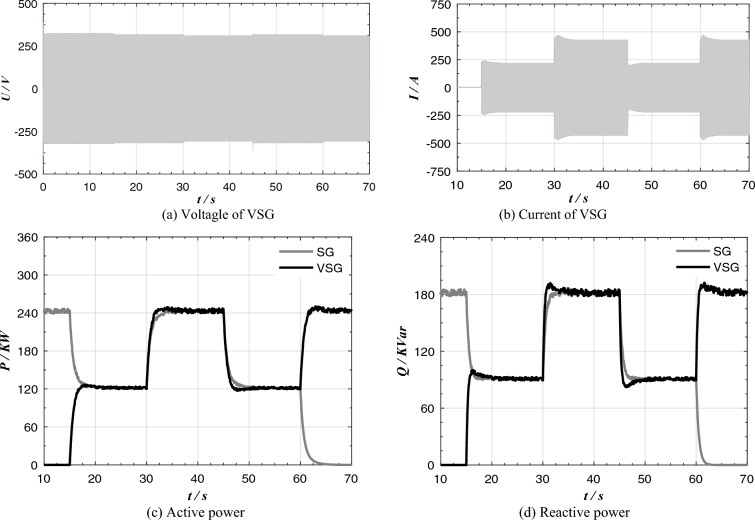
Experimental results of parallel operation of improved VSG and SG.

After adopting the improved control algorithm, the experimental results are shown in Fig. 12. Compare with the experimental results of the traditional VSG control algorithm in Fig. 11, the shock current of the system is decreased significantly, VSG and SG can better share the load in transient process, and the output power overshoot of VSG and SG is very small. At t = 15 s and t = 60 s, smooth switching between parallel operation and independent operation of heterogeneous power supply network can be realized.

**Figure 13:**
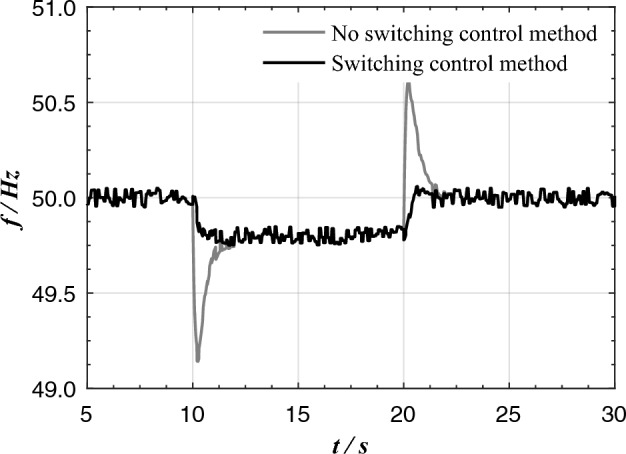
VSG independent running frequency dynamic feature with or without mode switching control.

If switching control is not designed and the algorithm with additional control link in parallel mode is still maintained when operating independently, the dynamic drop and rise of VSG frequency and the recovery time of frequency will be seriously affected. Figure 13 compares the frequency characteristics of VSG control algorithm with or without mode switching control under the conditions of sudden increase and sudden decrease of load in independent operation. The existence of additional links makes the dynamic overshoot of frequency reach 0.6Hz, and the frequency recovery time is about 2s. When switching to traditional VSG control, the frequency change has no overshoot, and the frequency recovery time is about 1.25s. It can be seen that the mode switching control not only ensure the fast dynamic response when VSG operates independently, but also realize transient power equalization between heterogeneous power sources when VSG operates in parallel with SG network.

## Conclusion

Differences between traditional VSG and SG in speed regulation, excitation and output impedance result in uneven transient power distribution between VSG and SG in isolated island microgrid. Based on the analysis of uneven transient power distribution and considering the fast dynamic response characteristics of VSG in independent operation, an improved transient power equalization control method based on mode switching is proposed. By introducing virtual speed regulation, virtual excitation and dynamic virtual impedance algorithm, the transient power of VSG and SG can be equally distributed in heterogeneous power supply parallel operation mode. Maintain traditional VSG control in independent operation mode to provide fast voltage regulation and frequency modulation capability. Smooth switching control based on state tracking is designed between the two modes. Hardware-in-loop experiment results show that the improved control method can significantly improve the transient power distribution between virtual synchronous generator and synchronous generator, and can smoothly switch between independent and networked parallel operation modes.

## Data Availability

All data generated or analysed during this study are included in this published article.
